# The choice and preference for public-private health care among urban residents in China: evidence from a discrete choice experiment

**DOI:** 10.1186/s12913-016-1829-0

**Published:** 2016-10-18

**Authors:** Chengxiang Tang, Judy Xu, Meng Zhang

**Affiliations:** 1 School of Public Administration, Southwestern University of Finance and Economics, Chengdu, Sichuan 610072 China; 2 Medicine College, Fujian Medical University, Xue Yuan Road, University Town, FuZhou, Fujian 350108 China

**Keywords:** *Health care utilisation*, *Private hospital*, *Discrete choice experiment*, *Hukou*, *China*

## Abstract

**Background:**

Public health care dominated the services provision in China before 1980s. However, the number of private health care providers in China has been increasing since then. The growth of private hospitals escalated after a market-oriented reform was implemented in 2001. Through an experimental approach, this study aims to a better understanding of the dynamic change in preference of health care utilisation among the residents in urban China.

**Methods:**

Based on a discrete choice experiment (DCE) from a random sample of respondents in urban China, the study evaluated preference over health care attributes affecting individuals’ choice for the utilisation of hospital health care. The marginal willingness-to-pay for five health care attributes was estimated, including public/private provision of health care, by analysing mixed logit and latent class models.

**Results:**

The results indicated a significantly negative marginal willingness-to-pay for private health care, which was interpreted as representing people’s previous interactions with the health care system. The latent class model further suggested preference heterogeneity across our sample. We found that Hukou type, a typical indicator of socioeconomic background, was significantly related to respondents’ preference for health care utilisation. Permanent urban residents (urban Hukou) valued private health care less; in contrast rural migrants (rural Hukou) were more likely to be indifferent between public/private provision.

**Conclusion:**

Urban residents in China showed a high disposition to obtain health care from the public providers of health care. Our results have implications in the context of the Chinese government attempts to expand the private health care sector in the short term. Policy makers need to consider residents’ preference for health care in health policy development as the preference can only change in the long term.

**Electronic supplementary material:**

The online version of this article (doi:10.1186/s12913-016-1829-0) contains supplementary material, which is available to authorized users.

## Background

The health care system in China has been dominated by hospital institutions, especially the public hospitals. Hospitals, particularly public hospitals, provide ninety percent of the country’s inpatient and outpatient health care services [1]. However, as a part of the consequence of public financial stagnation and decline beginning in the early 1990s, public health care systems in China have suffered from severe revenue shortfalls during a time when the population is getting older and richer. Inevitably, quality has deteriorated in public hospitals.

Faced with a rise in the demand for health care and a long waiting-list in public hospitals, the government has applied a series of market-oriented policies to endorse the development of a private hospital sector since 2000, which has resulted in a rapid increase in the number of private hospitals in China [2, 3]. More and more people choose to obtain health care services from the private hospitals, especially those patients with middle-low income [4]. However, there is still limited information on the demand side in the literature, specifically how individuals choose health care services, and whether or not they consider the characteristics of the providers in their choice.

Most of the published literature investigating the demand for private health care providers among the range of provider choices is from high-income settings, although we are aware of some studies completed in middle-or-low-income countries [5–7]. In general, patients in the US prefer non-profit and private health care providers, whereas the UK’s patients prefer public hospitals over private ones [8–10]. As we know, however, health care systems in developed countries are significantly different from those in developing countries. For example, the private hospital sector in China is substantially underdeveloped in contrast to it in developed countries [1].

The existing literature on the demand for health care in China is scarce and limited, with the focus being on the overall utilisation level or probability [4, 11]. Previous studies rarely control for the biases related to various types of illnesses [12, 13]. Moreover, the majority of studies find significant preferences for contextual factors (e.g. social and demographic characteristics), the perceived need for care (e.g. perceived characteristics of perceived illness), and enabling factors (e.g. access to health care) [14–16]. The public/private feature of health care has been examined in one study, in which the chance to work in the private health sector is valued by doctors, however the impacts of public/private attribute on patients are still unknown in current literature [17].

Although there is a wealth of literature on determinants of provider choice for health treatments, most studies analyse the health care seeking decision ex post. Studies typically make use of observational data or revealed-preference data and analyse which patients’ characteristics may explain heterogeneity in health care utilisation [4, 18]. To the best of our knowledge, only a few of the studies have used discrete choice experiments (DCE) in research on health care demand in developing countries. For example, Hanson and his colleagues explored the preferences for hospital quality in Zambia with the results from DCE, yet the attribute of hospital type was not considered [19].

With this paper, we try to contribute to a better understanding of the health care utilisation in developing countries. Specifically, we investigated whether Chinese urban residents prefer the public sector’s health care provision in a developing country with rapid growth of private health sector. Our study first takes advantages of a discrete choice experiment to evaluate the attributes of health care, including public/private provision of health care, based on a random sample of respondents in urban China. We apply conditional logit, mixed logit and latent class models to analyse preference heterogeneity for specific attributes of health care. Our results demonstrate that there is significant heterogeneity in the valuation of the attributes, and this heterogeneity is significantly correlated with Hukou type, a typical indicator of socioeconomic background in China [20, 21].

We further estimate the marginal willingness-to-pay (MWTP) for five health care attributes: knowing the doctor, the hospital type, the travel time, the waiting time, and the number of visits via the latent class random utility model. The analysis indicates that respondents are willing to pay a higher value for public providers of health care than the private one, which can be interpreted as representing their past experiences with the health care system. This finding is consistent with a previous study [22], which qualitatively reported rural-to-urban migrants in China commonly using private health care both before and after migration. The results of the stated preference experiments that focus on provider traits are highly complementary to insights from previous studies that focus on patients’ characteristics. Our finding has substantial policy implications in the contexts that China’s government made an ambitious initiative to attract private capital into health care industry before 2017 [23, 24]. We suggest that it is important to take into account the public preferences on the public before any decision to expand the private sector.

## Methods

Discrete choice experiment is one of the popular stated preference methods in health economics [25–27], which can be used to address a number of policy-related questions [28]. Adopting random utility theory, consumer theory and experimental design theory, the DCE method defines a hypothetical good or alternative (for example, health care service in this case) in terms of various attributes, in which individuals’ valuation will depend upon the different levels (or values) of these attributes [29]. A discrete choice experiment can be described by the following stages: (1) selection of attributes and levels, (2) experimental design and construction of choice sets, (3) survey implementation [26].

### Attributes and levels selection

Qualitative methods are suggested to develop the attributes and levels used in the DCE [30]. In this study, we first identified the conceptual framework based on previous studies that explored health care utilisations [4, 19, 31, 32]. To further refine the conceptual dimensions and develop general wording in DCE, we conducted two mini focus group discussions (less than 6 participants for each) among health care policy-makers and researchers in Australia and China respectively. All discussions were conducted by a moderator, audio recorded, transcribed and analysed in constant comparison approach. After exclusion of medicine availability from primary conceptual framework, six attributes from the qualitative study were included in the final DCE. The alternatives to hospital health care in the experimental choice design were considered to be appropriately characterised by six attributes. The rationale for selecting six attributes and their level are presented below:

1. Knowing the doctor: This attribute refers to the fact that the patient knows who their doctor is. Three levels are specified for this attribute—the unknown, the known, and the known well. This attribute may reflect patients’ information on doctors and medical knowledge [31].
2. Hospital type: The levels for hospital type are determined by the current provision situation within China—A-level Public Hospital (e.g. large teaching hospital), C-level Public Hospital (e.g. small community hospital), and Private Hospital. The provider attribute is included in the DCE design so that we can examine respondents’ preference for public and private hospitals [4].
3. Distance: Distance is represented by the travel time it takes for the respondent to visit the hospital to try to obtain health care. Travel time is respectively 20, 40 and 60 min on a one-way bus; since the public bus is the primary mode of transportation in China. This is a part of time costs and also an implicit out-of-pocket expenditure for some respondents [32].
4. Waiting time: This attribute indicates the time that a patient has to wait from registration until being diagnosed by the doctors, in which a psychological cost due to the anxiety of a health problem is included. Waiting time is respectively 0.5, 2 and 4 h, which is supposed to be consistent with previous research and statistics [33].
5. Out-of-pocket cost: This is the cost shared by the patient that represents the total charge by the hospital, minus the insurance refunds. These numbers are calculated using administrative health insurance claims data from local health departments. This is the only monetary attribute that can be used for estimating willingness-to-pay for other attributes.
6. Number of visits: The number of visits indicates length of the whole treatment. The more visits required, the more severe the illness and the higher explicit and implicit costs to a patient. The number of visits is assumed to be 1, 2 or 3, for either of the alternatives [32] Table 1.

**Table 1:** Attributes and levels for the DCE and related variables in estimation

Attribute	Definition	Attribute Levels	Variables
Knowing Doctor	Whether patients know who their doctor is	Unknown ^a^	
		Known	Known
		Acquainted	Acquainted
Hospital Type	Three major hospital choices	A-level Public (Large) ^a^	
		C-level Public (Small)	Small public
		Private Hospital	private
Distance	Distance from home to hospital (on bus)	>20min ^a^	
		>40min	min40
		>60min	min60
Time	Waiting time in hospital	= < 0.5h ^a^	
		= < 2h	hrs2
		= < 4h	hrs4
Out-of-pocket Cost	The amount of money paid by patient (RMB)	150 RMB($25)	
		300 RMB ($49)	
		450 RMB ($74)	
Number of Visit	The number of visits needed to have the required treatment	One ^a^	
		Two	Two
		Three	Three

Conversion rate is USD1.00 = RMB 6.16, www.oanda.com, June 23, 2014

^a^ Baseline level

### Experimental design

A full factorial design involves 729(3^6) possible combinations for each choice alternative. We created this DCE design using an orthogonal array (a fractional factorial design), in which a minimum of 18 choice sets were required in order to analyse the main effects, assuming that no significant interactions existed. The basic design was obtained from the mix-and-match method that was described in Louviere’s book [34], for an unlabelled design. These 18 choice sets were further divided into two blocks so that each respondent only needed to answer 9 questions in the experimental part of the questionnaire. In each scenario, respondents were asked which hospital they would choose between “Hospital Service 1” and “Hospital Service 2”, if they or their family member had a bone fracture condition. Everything about the hospitals’ health care being compared was the same, except for the varied attributes in alternatives.

**Fig. 1:**
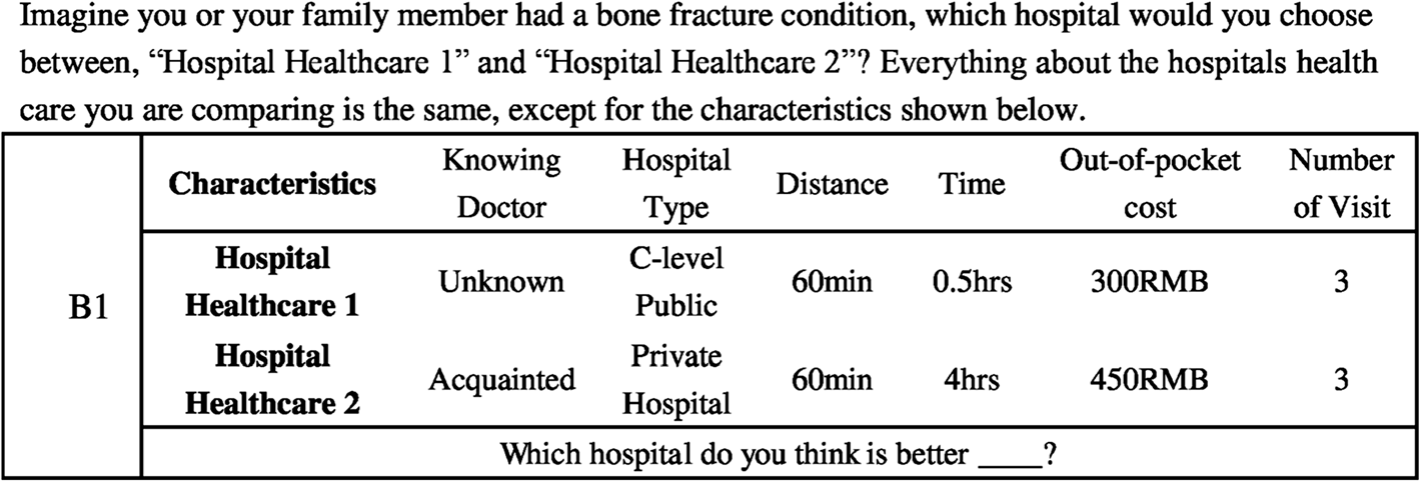
Sample choice set

This DCE design with two unlabelled choice options, excluding an opt-out option, was considered to be realistic in the case of the Chinese health care system for several reasons. First, an unlabelled format aims to encourage respondents to focus on the variation of attribute levels in each alternative, instead of the label of the alternative [25, 35]. An unlabelled design can also be helpful in minimising the number of required choice sets. Second, we chose two alternative designs without an opt-out option because this appeared to reduce respondents’ cognitive burden, even though a couple of papers suggested that an unforced choice would be preferred [28, 36]. Third, we selected a bone fracture condition in the hypothetical scenario because this is a common case for all families, and often sufficiently serious enough to visit a hospital immediately. Therefore, it was consequently possible to obtain real preference expressions from a large sample of the adult population Fig. 1.

### Data collection and analysis

The questionnaire applied in the survey included two parts: in the first part, respondents were asked about a set of demographic and socioeconomic questions that were modified from a published survey on primary health care in the same city [37]; the second part involved 9 DCE choice sets that were divided by the 18 minimum required choice sets so that two blocks of the questionnaire needed to be prepared. (See Additional file 1 for the questionnaire).

Ten well-trained undergraduate students from a local medical university conducted all of the interviews, in which the interviewers were responsible for explaining the survey, going through the questionnaire and collecting responses from respondents. Training included understanding the DCE design and questions, identifying people who were qualified to participate, eliciting and recording the responses. The training course introduced three common biases,^1^ such as information bias, non-trader bias and strategic bias [38], in DCE data collection, and taught interviewers how to prevent biases by probing and motivating respondents to make trade-off in decision-making.

Data collection was carried out in Fuzhou of Fujian province, where a developing private hospitals market provides an appropriate context for this study [3]. In order to cover both urban residents and rural-urban migrants, we surveyed three local markets in three different communities. The one is a downtown community where the majority of residents are the urban residents; the second is a suburban community inhabited by rural-urban migrants; the third is located at an urban-rural fringe area which is a mixed community. Five hundred and seven respondents who were over 18 years of age were randomly recruited at the market after receiving their informed consent. The sample size was based on the most commonly cited rule-of-thumb proposed by Orme, who gave an equation^2^ to determine a minimum sample size required for DCE involving the main estimation effects only [39].

We started by estimating a conditional logit model (CL) that was based on the random utility theory [40]. In addition to the conditional logit model, we further carried out both a mixed logit model (MXL, or random parameters logit model) and a latent class model (LCM, or finite mixture analysis) as approaches to account for heterogeneity in the preferences among respondents.^3^ Calculating marginal willingness-to-pay (MWTP) or implicit prices that follow the standard utility difference expression is a more convenient way to interpret coefficients in a mixed logit model and latent class model [41]. MWTP represents the marginal utility associated with a change in a health care attribute in monetary units, which can indicate how much an individual would be willing to pay to take or avoid a particular change in a given health care attribute.

## Results

### Description

Table 2 presents the summary statistics of our sample of 507 respondents. The average age of respondents was 32 in our sample, which is younger than the estimated average age (34) for urban and rural residents in local areas according to the Six Nationwide Population Census in China [42]. The percentage of male respondents accounted for 58.78 % of participants, while the average ratio of males in the local region is 51.45 %. About 47.73 % of respondents registered as urban households, while the percentage of those who registered in rural areas is 57.09 %. The distribution of education level is skewed to the right, with 35.5 % respondents having a university degree. The mean monthly income of respondents participating in our survey was about 3014 CNY, compared to the average monthly per capita income of 3258 CNY in 2014. The income is reasonable if we take rural-urban migrants in our sample into account. Of 507 valid responses, 56 % of the surveyed individuals were working in full-time jobs; 28.8 % were retired or students; around 10 % of participants were not specified. Our sample mainly covers the original urban residents and rural-urban migrants, so it is reasonable to find out more male and educated respondents in our sample than general population [43]. To guarantee the representativeness of our data, we must employ a random sampling method to collect our data. As an interviewer-administered survey, the sample can only be broadly representative of the urban population in the city.

**Table 2:** Descriptive information for respondents

	Block 1	%	Block 2	%	Total	%
Age	30.98 (12.19)		33.23 (14.33)		32.11 (13.34)	
Gender						
Male	146	57.71	152	59.84	298	58.78
Female	107	42.29	102	40.16	209	41.22
Household registration						
Urban registration	120	47.43	122	48.03	242	47.73
Rural registration	133	52.57	132	51.97	265	52.27
Education						
Junior middle school or below	49	19.37	67	26.38	116	22.88
High school	41	16.21	47	18.50	88	17.36
Vocational Diploma	62	24.51	61	24.02	123	24.26
University	101	39.92	79	31.10	180	35.50
Monthly income						
< 1001 RMB ($162)	72	28.46	75	29.53	147	28.99
1001–3000 RMB ($162–$487)	70	27.67	76	29.92	146	28.80
3001–5000 RMB ($487–$812)	73	28.85	66	25.98	139	27.42
> 5000 RMB (>$812)	38	15.02	37	14.57	75	14.79
Employment						
Employed full-time	151	59.68	133	52.36	284	56.02
Employed part-time	12	4.74	19	7.48	31	6.11
Not employed	1	0.40	6	2.36	7	1.38
Retired or Student	68	26.88	67	26.38	135	26.63
Other (Specify)	21	8.30	29	11.42	50	9.86
Total	254	100.00	253	100.00	507	100.00

Conversion rate is USD1.00 = RMB 6.16, www.oanda.com, June 23, 2014

mean coefficients; sd in parentheses

### Estimation results

Table 3 illustrates the regression results for conditional logit model and mixed logit model. A total of 4563 choice sets were included in the estimation (507 respondents, 9 choice sets each). In general, all coefficient estimates were consistent across the conditional logit and mixed logit models. The signs of parameters can be compared, however, the magnitude of coefficient estimates were not able to be compared.

**Table 3:** Estimated results for attributes levels in DCE

	Conditional Logit	Mixed Logit	
	Mean Coefficients	Mean Coefficients	SD
Small public	−0.465***	−0.509***	0.184
	(0.0622)	(0.0735)	(0.300)
Private	−0.990***	−1.257***	0.866***
	(0.0893)	(0.110)	(0.125)
hrs2	−0.247	−0.237	0.499
	(0.139)	(0.324)	(1.042)
hrs4	−0.998***	−1.339***	−0.998***
	(0.120)	(0.183)	(0.280)
Known	−0.0529	−0.0358	−0.0128
	(0.0531)	(0.0664)	(0.111)
Acquainted	0.0381	0.0582	0.556***
	(0.0756)	(0.0863)	(0.104)
min40	−0.606***	−0.706***	−0.0536
	(0.0675)	(0.0797)	(0.133)
min60	−1.053***	−1.277***	−0.174
	(0.0971)	(0.113)	(0.212)
Two	−0.287***	−0.376***	0.0195
	(0.0753)	(0.0912)	(0.137)
Three	−0.521***	−0.683***	0.778***
	(0.0739)	(0.0938)	(0.112)
Cost	−0.00189***	−0.00243***	
	(0.000218)	(0.000274)	
Asc	0.184***	0.236***	
	(0.0338)	(0.0412)	
N	4563	4563	
Pseudo R-sq	0.155		
ll	−2671.2	−2629.7	
chi2		82.99	
df_m		10	

Standard errors in parentheses

*** *p* < 0.001

The first column shows the health care attribute, and the other columns present coefficients^4^ and standard errors in parentheses. Comparing with a large public hospital (the baseline level), an alternative with the small public hospital (−0.465) decreases the likelihood of choosing an option, as does even worse for the private hospital (−0.990). This result suggests that patients in China prefer large and public hospitals over private hospitals and small-public hospitals. As expected, the higher the waiting time in hospital (−0.998 for 4 h), the less likely a patient was to choose that particular alternative, but it appears that a short waiting time, like 2 h, revealed no difference compared to the baseline level—the half hour. However, the coefficients for the attribute of knowing the doctor were not significant, since we expected that the information about the doctor would increase the possibility to select that option.

In the MXL model, we assigned a normal distribution to five health care attribute parameters, and the variable out-of-pocket cost was specified as non-random. Thus, the preferences for the price of the health care were assumed to be homogeneous, that is, the marginal utility of price was assumed to be constant over the sample in order to calculate MWTP. Additionally, the large and significant standard deviation estimates in the MXL model indicate the presence of considerable preference heterogeneity, which suggests the application of a latent class model. For example, comparing the mean effect of − 1.257 for the private hospital to large public hospital to a standard deviation of 0.866 tell this relationship.

In Table 4, we identified the optimal number of classes in LCM by assessing the Bayesian Information Criterion (BIC), the Consistent Akaike Information Criterion (CAIC) and the log likelihood statistics of the models with 2–5 classes [44]. Both BIC and CAIC implied that their minimum value were at the 2-class model, which suggested the unnecessary to search for higher classes [27]. Meanwhile, we used the Akaike likelihood ratio test to compare the MXL and LC models [45]. We followed examples of other choice experiment papers to compare both conditional logit models with the MXL and LC models using a likelihood ratio test [46, 47]. Overall, LC was recommended as the preferred model in this analysis, as accounted for both observed and unobserved heterogeneity in comparison with both the conditional logit model and MNL model.

**Table 4:** Selection criteria of latent classes

Classes	LLF	Nparam	AIC	CAIC	BIC
2	−2520.981	30	5101.962	5258.4	5228.4
3	−2477.178	48	5050.356	5300.658	5252.658
4	−2445.957	66	5023.914	5368.078	5302.078
5	−2423.697	84	5015.393	5453.421	5369.421

As can be seen from the upper section of Table 5, the two preference classes have a clear interpretation: Class 1 was more likely to select a private hospital compared to large public hospitals, while Class 2 was more likely to choose large public hospitals. We labelled our classes accordingly, as non-public-oriented and public-oriented. Class 1 had an average membership probability of 21.1 %, and Class 2 of nearly 78.9 %. The two classes reflected the opposite attitudes vis-à-vis the relative importance of attributes. The members of non-public-oriented class did not discriminate against private hospitals when they made a choice on health care; on the contrary, the members of the public-oriented class obtained higher utility when the large public hospital was included as one of dimensions in the attribute. In despite of hospital type, all other coefficients for DCE attributes were consistent across two classes. While we look at the coefficients of variables that determined the membership of classes, we found only “Hukou”, among all socio-economic variables included in the estimation, had an impact on choice. Respondents from rural households, *ceteris paribus*, are more likely than those from urban households to be non-public-oriented members.

**Table 5:** Estimated results for attributes levels in DCE (Latent class model with 2 classes)

	Non-public-oriented class	Public-oriented class	Co-variables
Small public	0.268	−0.604***	
	(0.306)	(0.124)	
Private	0.348	−1.278***	
	(0.422)	(0.166)	
hrs2	0.480	−0.198	
	(0.721)	(0.193)	
hrs4	−1.454***	−0.911***	
	(0.441)	(0.161)	
Known	0.562*	−0.0721	
	(0.262)	(0.0807)	
Acquainted	0.488	0.0672	
	(0.332)	(0.106)	
min40	−0.767*	−0.723***	
	(0.324)	(0.0925)	
min60	−1.122**	−1.168***	
	(0.391)	(0.125)	
Two	−0.920**	−0.112	
	(0.294)	(0.104)	
Three	−2.129***	−0.212*	
	(0.552)	(0.100)	
Cost	−0.00327**	−0.00168***	
	(0.00115)	(0.000286)	
Asc	0.644***	0.110*	
	(0.124)	(0.0471)	
Age			−0.0263
			(0.0228)
Gender			0.140
			(0.403)
Income			0.318
			(0.215)
Edu			0.223
			(0.188)
Hukou			1.623**
			(0.496)
Work			−0.188
			(0.136)
_cons			−4.384*
			(1.859)
Percentage	21.10%	78.90%	
N	4563		

Standard errors in parentheses

* *p* < 0.05

** *p* < 0.01

*** *p* < 0.001

### Marginal willingness to pay

The comparison of coefficients among three models is not available, as models are parameterised in different ways [27], however implicit prices or the marginal willingness-to-pay can be calculated for each of the attributes for three reported models. In Table 6, we report the mean marginal WTP for each attribute for the CL, MXL and LC models. The mean estimates of attributes differ slightly across the three models, especially for the first two models, the conditional logit model and the mixed logit model. We focused on the WTP in LCM. We found that the weighted average marginal WTP of segments for increasing waiting time, distance, and visits were RMB − 62.17,−522.05,−389.32,−621.63,−112.07, and − 236.93, respectively; where the negative sign for waiting time, distance, and visits reflected that respondents on average are willing to pay in order to improve the accessibility to health care. However, the mean WTP for hospital type between two classes was totally opposite, which proved that individuals in two segments have different tastes when choosing public and private hospitals.

**Table 6:** Marginal willingness-to-pay for each attribute across models (RMB)

	CL	MXL	Latent class model		
			Segment 1	Segment 2	Weighted average of segments
Small public	−246.23	−209.49	*81.84*	−359.55	−266.42
Private	−524.90	−517.09	*106.50*	−761.11	−578.04
hrs2	*−130.74*	*−97.53*	*146.59*	*−117.99*	−62.17
hrs4	−529.06	−550.61	−444.43	−542.81	−522.05
Known	*−28.02*	*−14.71*	171.72	*−42.96*	2.34
Acquainted	*20.21*	*23.94*	*149.27*	*40.04*	63.09
min40	−320.90	−290.33	−234.58	−430.70	−389.32
min60	−558.23	−525.31	−343.00	−696.14	−621.63
Two	−151.86	−154.66	−281.12	*−66.87*	−112.07
Three	−275.97	−281.05	−650.67	−126.29	−236.93

The numbers in italic refers to insignificant estimates; otherwise the parameters are significantly different from zero at a 1% level of confidence

### Validity of DCE

There are two important assumptions for the validity of Discrete Choice Experiments. The first is whether the respondents can understand the structure of the DCE, and thus make rational decisions in choice sets. The other is the appearance of dominant preference for one or a couple of specific attributes in which a lack of trade-off between attributes would result in the unavailability of the indifference curve and marginal rate of substitution. A consistency test is conventionally conducted to deal with the first problem, yet we did not perform it by including a choice set in which one alternative is obviously preferred to the other. As in our DCE, the face-to-face interview by an experienced surveyor and a small number of questions (only 9 questions) for each respondent largely mitigated the necessity for a consistency test. We performed the Dominance preferences test in which a pseudo t-test is examined by comparing a specific attribute parameter estimated in the full model including all variables with the same attribute parameter estimated in the reduced model including only this attribute [48]. It is the indication of dominant preference for this attribute if the null hypothesis for the two parameters (β_i_ = β_j_) cannot be rejected. Our tests did not support the argument that a dominant attribute existed in this DCE.

## Discussion

This study identified a range of attributes that influence preference for health care utilisation that are relatively familiar to respondents, including knowing the doctor, hospital type, distance from the hospital to the residence, waiting time in the hospital, out-of-pocket cost and the number of visits during the whole treatment period. In the design of our experiment, we chose attributes that could represent a suitable range of the cost and quality dimensions of the health care through the focus group discussions. The purpose was to minimise the unmeasured preference variation between the alternatives, and also to some extent avoid the dominant preference as the DCE study assumes independent attributes to investigate the main effects by estimation.

Our results show that public/private provision of health care is an important attribute taken into account when individuals are searching for health care in China. We further suggest that this particular attribute can be interpreted as reflecting respondents’ previous experiences with the health care system in China. Past interactions with the health care system have been considered as an influential factor in health care utilisation in the literature [49]. One important result in our study is that respondents generally prefer public health care. Additionally, the other three identified attributes—distance from hospital to residence, waiting time in hospital, and the number of visits during the whole treatment period—are in the spirit of the most theoretical models of the health care utilisation. As the majority of Chinese residents, no matter in rural or urban area, are covered by one of three public health insurances at present, the out-of-pocket cost is included in order to highlight the real cost for a specific illness. Previous papers in Gambia and UK also considered drug availability, examination, parking facilities, and complications from treatment which did not seem to be feasible factors in our context [50, 51].

On the other hand, this study examined the impact of preference heterogeneity on the estimates. The LCM captures the variation in preferences for specific attributes, and thus helped us to identify public-oriented and non-public-oriented classes. The first class, labelled public-oriented respondents comprised 79 % of the sampled individuals. They value more public health care than private health care, and they are basically urban Hukou. In contrast, our analysis identified nearly 21 % of sampled respondents as non-public-oriented individuals, who derive similar utility from either public or private health care. Respondents in this class more likely have rural Hukou. Our results are basically consistent with Goodburn’s qualitative findings, in which she found rural-to-urban migrants in China commonly use private health care both before and after they move to an urban area [22].

Why is utility for private hospitals lower? Why don’t residents with rural Hukou discriminate against private hospitals? It is reasonable in a sense, but beyond expectation that notorious Hukou or the household permanent residence registration system in China works in shaping residents preference for health care access. We provide two explanations: one possible explanation for this result is that private health care in China is cheap and often with lower quality, Tang showed that fees in private hospitals are lower for a specific illness when other conditions are the same [52]. Second, the Hukou determines health insurance rights and thus, the access to health care. There are sharp restrictions on changing from a rural to non-rural Hukou. Nowadays, people from rural areas can now move to urban areas in order to work with temporary mobility permits. However, as these migrants still hold a rural Hukou, they do not have access to the urban health insurance that to a degree is related to the hospital health care in urban areas. So our results show the Hukou limitation does have an impact on health care access among urban residents in China.

This study has substantial policy implications in the contexts that China’s government attempts to increase market share for private hospitals from 10 % to 20 % before 2017 [23, 24]. In terms of marginal willingness-to-pay, the estimates suggest that the option of being able to choose their care in a public hospital would be valued at a price of over RMB 500, all else being equal. In the current context, the choice of the public hospital might be reflecting a more general support for the public health system as a whole. One important implication is that it is often rather difficult to change the provider preference among urban residents than health policy makers may think. As mentioned above, public provision of health care in China has a long and robust history.

Additionally, two potential consequences may be raised regarding the policy target, the one is private investors are not willing to invest enough capital given limited demands for the private health care. The other is that the rapid expansion of the private investment fails to provide appropriate services, which further damage the private’s reputation and makes it harder to change residents’ taste for the private health care. Another policy implication is that it is unacceptable politically to sustain a rural-urban separated health care system that brings up urban residents with higher taste for health care. It is better to design policy incentives in order to influence residents’ preference about public-private dimension of health care providers. We thus provide a clear example of where understanding preferences is important for effective health care policy making.

There are a few of limitations we must be open to acknowledge in this study. In the case of experimental design, there were a number of constraints including the lack of use of the status quo option, and the lack of prior information for use in generating the experimental design. To elicit preference with more confidence, a consistent test or some more comprehensive techniques, such as best-worst choice, should be included in the questionnaire in future studies. In addition, our sampling strategy, like any interviewer-administered survey, may also suffer from selection bias. Even though the sample was broadly representative of the population in local urban areas, the sample of 507 respondents for this data was slightly more educated than the general population, and had additional males than the general population.

## Conclusion

In conclusion, this paper first applied a discrete choice experiment to value the attributes of health care, including public/private provision of health care, in the context of growing private hospitals in China. The respondents in urban China showed a high disposition to obtain health care from the public providers of health care, which may reflects their previous interactions with the health care system. We also found an evidence of preference heterogeneity that is correlated with respondents’ typical socioeconomic background in China. Specifically, permanent urban residents (urban Hukou) value private health care less; in contrast, rural migrants (rural Hukou) are more likely to be indifferent between public/private provision. Our results have implications in the context of the Chinese government attempts to expand the private health care sector in the short term. Policy makers need to consider residents’ preference for health care in health policy development as the preference can only change in the long term.

## Additional file

Additional file 1: Questionnaire. (DOCX 24 kb)

## Footnotes

1 Information bias refers to respondents may not be aware of all alternatives in a choice set, or may make associations with other alternatives that are not included. Non-trader bias refers to respondents did not make trade-off between the values of alternatives. Strategic bias refers to respondents uses a heuristic way in choice decisions in which they did not compare all presented alternatives in a choice set simultaneously.

2 (*nta*)/*c* > =500, where *n* is the number of respondents, *t* is the number of tasks, *a* is number of alternatives per task (not including the none alternative), and *c* is equal to the largest number of levels for any one attribute.

3 CL model allows full covariance among alternatives using a joint normal distribution, in which it assumes that respondents have the same preferences (or that their preferences depend on observable characteristics). MXL model overcomes these limitations by allowing the coefficients in the model to vary across decision makers. Alternatively, the coefficients may be discrete, which leads to the LCM.

4 We interpret coefficients as the impact of the attribute’s difference between alternative 1 and alternative 2 on the probability of choosing one over the other. The positive (negative) sign of coefficients reflects whether the attribute level increased (decreased) the likelihood of the alternative to be chosen.
