# Supplementary material for: The choice and preference for public-private health care among urban residents in China: evidence from a discrete choice experiment
Source: BMC Health Serv Res. 2016 Oct 18;16:580. doi: 10.1186/s12913-016-1829-0 (PMC5070360; doi:10.1186/s12913-016-1829-0)
Supplement: Additional file 1: — Questionnaire. (DOCX 24 kb) [file 12913_2016_1829_MOESM1_ESM.docx]

A. Demographic/socioeconomic characteristics

| A1 | Are you 1 Male 2 Female |
| --- | --- |
| A2 | Your birth year __________ |
| A3 | Your monthly income: |
|  | 1 ≤1000 RMB 2 1001-2000 RMB　 3 2001-3000 RMB  4 3001-4000 RMB 5 4001-5000 RMB　 6 5001-10000 RMB 7 Above 10000 RMB |
| A4 | Your highest grad in school: |
|  | 1 Primary school or under primary school 2 Middle school　 3 High school 4 Professional High school 5 Technical secondary school 6 Technical secondary school 7 Diploma course 8 College 9 Master degree and above |
| A5 | Your household registration: 1 Urban Registration 2 Rural Registration |
| A6 | Are you: |
|  | 1 Employed full-time 2 Employed part-time 3 Not employed 4 Retired, Student 5 Other (Specify) ______ |

B. Imagine you or your family member had a bone fracture condition, which hospital would you choose between “Hospital Healthcare 1” and “Hospital Healthcare 2”? Everything about the hospitals healthcare you are comparing is the same, except for the characteristics shown below.

| B1 | **Characteristics** | Severity | Hospital Type | | Distance | | Time | | Out-of-pocket cost | | Number of Visit | |
| --- | --- | --- | --- | --- | --- | --- | --- | --- | --- | --- | --- | --- |
|  | **Hospital Healthcare 1** | Severe | Private Hospital | | 60min | | 4hrs | | 450RMB | | 3 | |
|  | **Hospital Healthcare 2** | Severe | Private Hospital | | 20min | | 0.5hrs | | 300RMB | | 2 | |
|  | *Which hospital do you think is better ____* | | | | | | | | | | | |
|  | | | | | | | | | | | | |
| B2 | **Characteristics** | Severity | Hospital Type | | Distance | | Time | | Out-of-pocket cost | | Number of Visit | |
|  | **Hospital Healthcare 1** | Mild | Private Hospital | | 60min | | 2hrs | | 150RMB | | 2 | |
|  | **Hospital Healthcare 2** | Severe | A-level Public | | 60min | | 2hrs | | 300RMB | | 1 | |
|  | *Which hospital do you think is better ____* | | | | | | | | | | | |
|  | | | | | | | | | | | | |
| B3 | **Characteristics** | Severity | Hospital Type | | Distance | | Time | | Out-of-pocket cost | | Number of Visit | |
|  | **Hospital Healthcare 1** | Moderate | Private Hospital | | 20min | | 2hrs | | 450RMB | | 1 | |
|  | **Hospital Healthcare 2** | Mild | A-level Public | | 40min | | 2hrs | | 450RMB | | 3 | |
|  | *Which hospital do you think is better ____* | | | | | | | | | | | |
|  | | | | | | | | | | | | |
| B4 | **Characteristics** | Severity | Hospital Type | | Distance | | Time | | Out-of-pocket cost | | Number of Visit | |
|  | **Hospital Healthcare 1** | Severe | A-level Public | | 公交60min | | 2hrs | | 300RMB | | 1 | |
|  | **Hospital Healthcare 2** | Moderate | C-level Public | | 40min | | 2hrs | | 300RMB | | 2 | |
|  | *Which hospital do you think is better ____* | | | | | | | | | | | |
|  |  | | | | | | | | | | | |
| B5 | **Characteristics** | Severity | | Hospital Type | | Distance | | Time | | Out-of-pocket cost | | Number of Visit |
|  | **Hospital Healthcare 1** | Mild | | C-level Public | | 60min | | 0.5hrs | | 300RMB | | 3 |
|  | **Hospital Healthcare 2** | Severe | | Private Hospital | | 60min | | 4hrs | | 450RMB | | 3 |
|  | *Which hospital do you think is better ____* | | | | | | | | | | | |
|  | | | | | | | | | | | | |
| B6 | **Characteristics** | Severity | | Hospital Type | | Distance | | Time | | Out-of-pocket cost | | Number of Visit |
|  | **Hospital Healthcare 1** | Severe | | Private Hospital | | 20min | | 0.5hrs | | 300RMB | | 2 |
|  | **Hospital Healthcare 2** | Mild | | Private Hospital | | 60min | | 2hrs | | 150RMB | | 2 |
|  | *Which hospital do you think is better ____* | | | | | | | | | | | |
|  | | | | | | | | | | | | |
| B7 | **Characteristics** | Severity | | Hospital Type | | Distance | | Time | | Out-of-pocket cost | | Number of Visit |
|  | **Hospital Healthcare 1** | Mild | | A-level Public | | 20min | | 0.5hrs | | 150RMB | | 1 |
|  | **Hospital Healthcare 2** | Severe | | C-level Public | | 40min | | 0.5hrs | | 450RMB | | 1 |
|  | *Which hospital do you think is better ____* | | | | | | | | | | | |
|  | | | | | | | | | | | | |
| B8 | **Characteristics** | Severity | | Hospital Type | | Distance | | Time | | Out-of-pocket cost | | Number of Visit |
|  | **Hospital Healthcare 1** | Severe | | A-level Public | | 40min | | 4hrs | | 150RMB | | 2 |
|  | **Hospital Healthcare 2** | Mild | | C-level Public | | 20min | | 4hrs | | 450RMB | | 2 |
|  | *Which hospital do you think is better ____* | | | | | | | | | | | |
|  | | | | | | | | | | | | |
| B9 | **Characteristics** | Severity | | Hospital Type | | Distance | | Time | | Out-of-pocket cost | | Number of Visit |
|  | **Hospital Healthcare 1** | Moderate | | A-level Public | | 60min | | 0.5hrs | | 450RMB | | 2 |
|  | **Hospital Healthcare 2** | Moderate | | C-level Public | | 60min | | 4hrs | | 150RMB | | 1 |
|  | *Which hospital do you think is better ____* | | | | | | | | | | | |
